# Missed Intra-Orbital Wooden Foreign Body

**DOI:** 10.7759/cureus.22078

**Published:** 2022-02-10

**Authors:** Sandip K Sahu, Priyadarshini Mishra, Anjali Kharolia

**Affiliations:** 1 Ophthalmology, All India Institute of Medical Sciences, Bhubaneswar, Bhubaneswar, IND

**Keywords:** intra orbital mass, diagnostic challenge, wooden foreign bodies, missed, intra orbital

## Abstract

Intra-orbital wooden foreign bodies (IOFB) are always a diagnostic challenge. Those are entered by unmarked trivial injuries, as in our case, may become even more difficult, and maybe notorious and remain quiescent for a long time, before presenting with a variety of complications. It may lead to a more diagnostic dilemma, which can especially occur with wooden foreign bodies (FB) due to the variable nature of radio-imaging. The lack of clinical suspicion may lead to a series of errors both in diagnosis and management, subjecting the patient to unnecessary intervention. In this case report, we will describe a missed wooden foreign body that, after a long quiescent period, presented as an intra-orbital mass.

## Introduction

Orbital disease or morbidity is always a diagnostic challenge for the ophthalmologist. Intra-orbital foreign bodies (IOFB) mainly enter the orbit between the eye and the orbital walls, but may occasionally enter the orbit through the eye or through the paranasal sinus into the orbit. Wooden foreign bodies (FB) enter through the eyes. Some unmarked trivial injuries, as in our case, may be notorious and remain quiescent for a long time before presenting with a variety of complications. There will be a diagnostic dilemma in missed foreign bodies as usual imaging and other diagnostic procedures did not detect intra-orbit wood due to their radiolucent nature. We report an unusual case of an intra-orbital wooden foreign body.

## Case presentation

A 70-year-old male presented with left eye mild pain, watering, discharges with restricted eye movement for six months. History-taking was unremarkable, except for the community bath. On examination, his vision was 20/40 with a senile cataract. The globe was intact, with mild ptosis, and restricted eye movement in all lateral gaze. Intra-ocular pressure (IOP) was normal, with regurgitation of pressure over the lacrimal sac.

Imaging was advised. A computed tomography (CT) scan of the left orbit in axial and coronal planes depicted a predominantly intraconal soft tissue density mass lesion measuring 2.5 (AP) × 1.5 (ML) × 1.5 (CC) cm^3^ involving the medial rectus and posterior aspect of the superior rectus muscle with a probable diagnosis of metastasis, lymphoma, or other mass lesion. Thus, a contrast study or magnetic resonance imaging (MRI) was advised.

**Figure 1:**
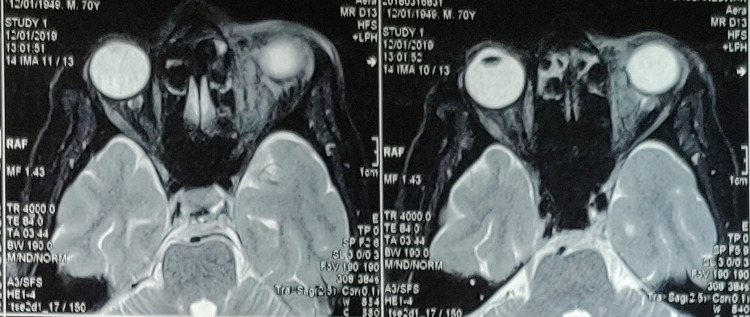
MRI orbital axial T2 image Hypointense lesion in left orbit involving the medial rectus and extending to postero-medial aspect of superior rectus abutting the lateral part of left optic nerve sheath

MRI revealed a well-defined T2 hypointense lesion in the left orbit involving the medial rectus and extending to the postero-medial aspect of the superior rectus, abutting the lateral part of the left optic nerve sheath. It was heterogeneously enhanced with contrast, pointing more towards an inflammatory origin (Figure 1).

**Figure 2:**
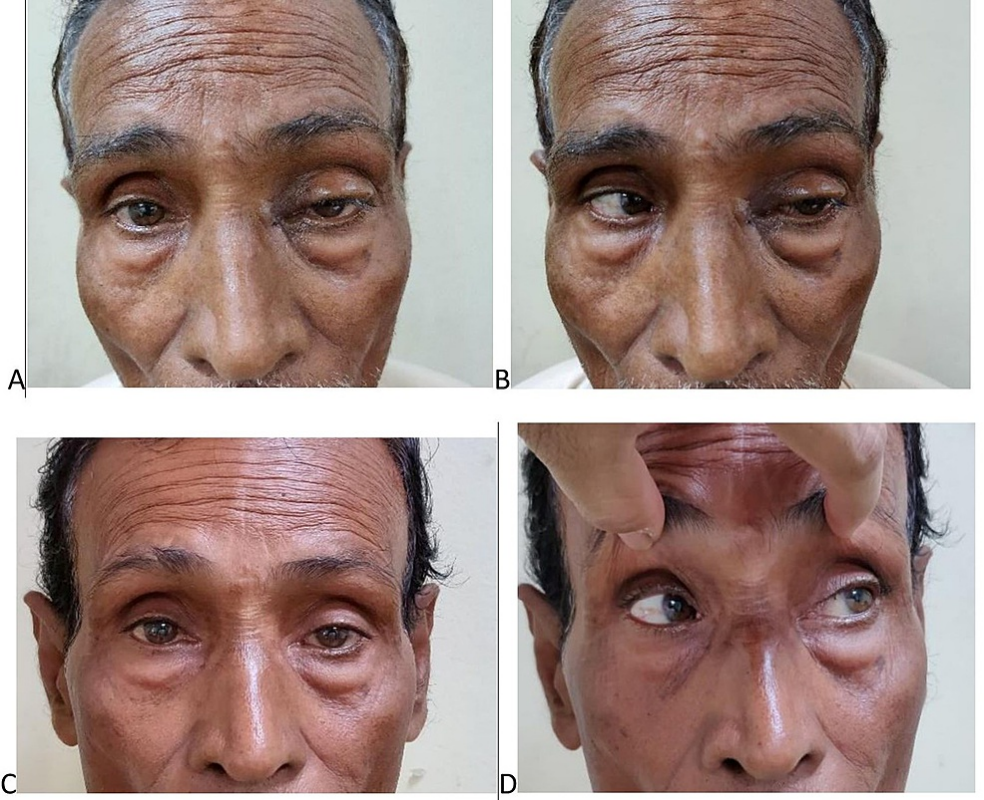
Pre and post-operative images (A, B) Pre-operative images of the patient in primary and lateral gaze and (C, D) post-operative images of the patient in primary and lateral gaze

**Figure 3:**
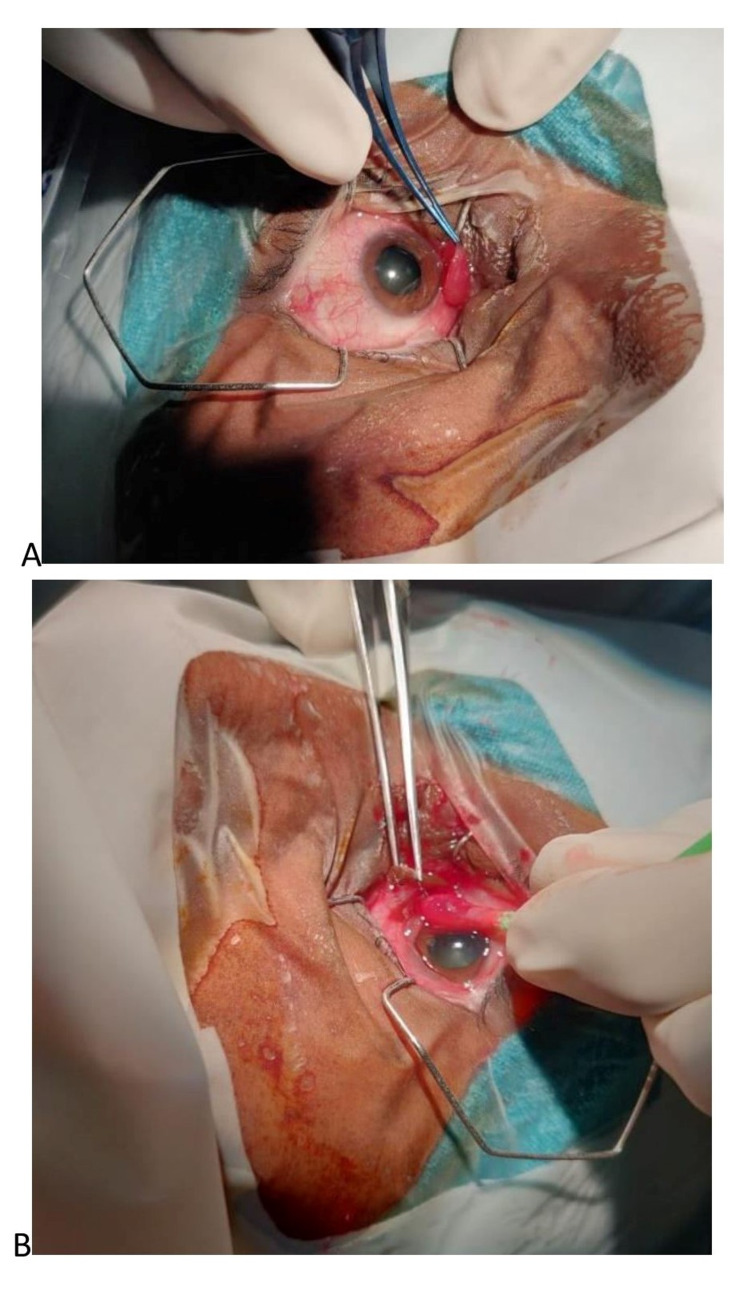
Intra-operative images (A) Inflammatory mass at medial canthus on bulbar conjuctiva and (B) wooden foreign body after dissecting the inflammatory mass

Routine blood investigations were within the normal limit. As the patient did not have systemic diseases or symptoms of any malignancies, he was given a course of oral steroids in a tapering dose and an oral antibiotic. Although the pain subsided, the ocular motility did not improve (Figure 2A, 2B). Meanwhile, the patient underwent dacryocystectomy. Surgery was uneventful, but on the seventh post-operative day, an inflammatory mass (Figure 3A) at the medial canthus of the bulbar conjunctiva lateral to the caruncle was noticed. This time, we planned an excisional biopsy. On table as we started dissecting to our utter astonishment, a black mass started peeping from behind. After removal, clinically it was confirmed to be a wooden foreign body (Figure 3B) measuring 2.0 × 0.2 × 0.2 cm^3^ and sent for histopathology and culture.

On examination, the biopsy report of the inflammatory mass revealed a section measuring 0.5 × 0.5 × 0.2 cm^3^ that shows fibrocollagenous tissue without any epithelial lining and contains edematous stroma, proliferated vascular channels with inflammatory cell infiltration consisting of predominantly plasma cells, lymphocytes, scattered eosinophils and neutrophils, and myofibroblasts seen. No evidence of granuloma, atypia or malignancy was seen in the section. There was no evidence of obliterative thrombophlebitis/fibrosis. On follow-up after two weeks, the restriction of ocular muscles had improved and the ptosis receded (Figure 2C, 2D).

## Discussion

Depending on chemical composition, IOFB can be classified into (1) metallic, (2) nonmetallic, and (3) organic [1]. Among the three types, organic substances are not only much more difficult to detect on imaging but also pose a higher risk of complications because secondary infection and decay of the FB may cause severe inflammation [2]. Among organic substances, wooden FB provides an ideal environment for microorganism growth due to its organic nature and porous consistency [3]. Complications like orbital cellulitis, abscess formation, chronic discharge sinus, extraocular muscle damage, exophthalmos, and central nervous system (CNS) infection can occur [4].

Our case was presented with mild pain, redness, discharge, and restricted ocular motility. As a result, the main differential diagnoses were infective or inflammatory orbital pathology. Lacrimal sac pathology was an additional finding. With proper clinical evaluation, the radiological examination also failed to identify the presence of the wooden foreign body. Post-operative repeated history of trivial injuries during a bath at the pond throws light on a wooden foreign body that may remain inconspicuous and yet be able to show its hazardous effects by acting as a natural reservoir of micro-organisms. The mere capability to camouflage orbital tissues and air in B-scan, plain X-ray, and CT scan, makes it arduous to diagnose [5]. Like in our case, it caused an intense granulomatous reaction [6] that not only compromised the function of the superior rectus and medial rectus but also the levator palpebrae superioris by its compression effect on the superior division of the ophthalmic nerve. The impression from the CT scan broadened our horizons to include lymphoma and metastasis in our differential diagnosis. The T2 weighted contrast-enhanced MRI (CEMRI) showing a heterogeneously hypointense lesion helped in narrowing the diagnosis to an inflammatory mass. It forced us to rethink that the trauma may be with wooden or organic materials, and subsequently, the patient was started on oral antibiotics, suspecting a foreign body might still be present inside.

## Conclusions

So, repeating leading questions to get a clue regarding the diagnosis is very essential. The normal appearance on the CT scan and USG does not rule out the possibility of a foreign body. At the same time, blind exploration of the orbit is not recommended for something suspected without positive proof of its position or even its existence. It is prudent to defer any surgical exploration until definite localizing signs become apparent. Such management offers a planned approach with minimal injury to orbital structures.
